# Granisetron-mediated augmentation of sertraline therapeutic effect in obsessive-compulsive disorder: a double-blind placebo-controlled, randomized clinical trial

**DOI:** 10.1186/s40360-022-00610-5

**Published:** 2022-09-27

**Authors:** Ala Ghobadian, Saba Mokhtari, Behnam Shariati, Leila Kamalzadeh, Mohsen Shati, Mehrdad Eftekhar Ardebili, Masoomeh Yarahmadi, Mohammadreza Shalbafan

**Affiliations:** 1 Mental Health Research Center, Psychosocial Health Research Institute (PHRI), Department of Psychiatry, School of Medicine, Iran University of Medical Sciences, Tehran, Iran; 2 Department of Psychiatry, University of Social Welfare and Rehabilitation Sciences, Tehran, Iran; 3 Mental Health Research Center (MHRC), School of Behavioral Sciences and Mental Health, Tehran Institute of Psychiatry, Iran University of Medical Sciences, Tehran, Iran; 4 Department of Epidemiology, School of Public Health, Iran University of Medical Sciences, Tehran, Iran; 5 Brain and Cognition Clinic, Institute for Cognitive Sciences Studies, Tehran, Iran

**Keywords:** Serotonin 5-HT_3_ receptor, Obsessive-compulsive disorder, Randomized controlled trial, Selective serotonin reuptake inhibitors, Granisetron

## Abstract

**Background:**

Medications currently recommended for the treatment of Obsessive-Compulsive Disorder (OCD) usually relieve the severity of symptoms by as much as 20–30%, and satisfactory treatment is obtained in 40–60% of patients with OCD. Nevertheless, the remaining symptoms continue to impair the patients’ function. Therefore, it is necessary to investigate possible strategies to improve the mitigation of symptoms. In this study, the main objective was to examine and investigate the effectiveness of granisetron, which is a serotonin 5-hydroxytryptamine receptor type 3 (5-HT_3_) antagonist, as an adjunct therapy to selective serotonin reuptake inhibitors, for the purpose of ameliorating OCD symptoms.

**Methods:**

fifty-eight patients diagnosed with OCD, based on Diagnostic and Statistical Manual of Mental Disorders (DSM-5) criteria, who had a Yale-Brown obsessive-compulsive scale (Y-BOCS) score of more than 21 were recruited in a double-blinded, parallel-group, placebo-controlled, clinical trial of 10 weeks to receive either granisetron (1 mg twice daily) and sertraline (100 mg daily initially followed by 200 mg daily after week 4) or placebo and sertraline. The primary outcome was OCD symptoms measured by the Y-BOCS.

**Results:**

Y-BOCS total score significantly dropped in both groups (28.9 to 17.7 for granisetron plus sertraline and 27.5 to 19.3 for placebo plus sertraline group with a slightly greater drop for granisetron plus sertraline group), while the granisetron plus sertraline group experienced a significantly greater reduction in obsession scores (Greenhouse-Geisser F(2.32,97.57) = 4.52,p-value = 0.01). Moreover, in comparison with the placebo plus sertraline group, the proportion of the patients showing complete response was considerably higher among the granisetron plus sertraline group (*P*-value < 0.01). No major adverse effects were observed in any of the groups.

**Conclusion:**

The results suggest that granisetron augmentation of sertraline may increase the rate of response in patients with moderate to severe non-refractory OCD. Further studies are suggested in this regard.

## Background

Obsessive-Compulsive Disorder (OCD) affects nearly 1–3% of the world population [1, 2]. OCD is characterized by intrusive thoughts which cause discomfort, apprehension, and/or repetitive behaviors which try to reduce the associated anxiety[3]. If left untreated, the illness trajectory is waxing and waning chronically [4]. OCD severely impairs quality of life and causes impairment in social and occupational function in patients[5, 6].

Currently, selective serotonin reuptake inhibitors (SSRIs) and/or cognitive behavioral therapy (CBT), particularly exposure and response prevention (ERP), are considered first-line treatments for OCD[7, 8]. SSRIs usually reduce the effect and severity of OCD symptoms by as much as 20–30%[9]. Satisfactory treatment is obtained in 40–60% of patients with OCD[10, 11]. Nevertheless, the remaining symptoms continue to impair the patients’ function. For patients who do not respond adequately to first-line treatments, combination therapy of CBT and SSRIs, pharmacological augmentation of SSRIs, and changing SSRIs to another drug (in case there is a specific SSRI resistance ) are used as second-line therapies[12].

Granisetron is a highly selective and potent 5-HT_3_ receptor antagonist which has very little or no affinity for other receptors.[13]. It is used to effectively prevent post-chemotherapy, post-radiotherapy, or post-surgery nausea and vomiting. Due to its high lipophilicity, granisetron is an ideal choice to pass the blood-brain barrier and to be used in nervous system disorders[14].

Even if, some previous studies have shown positive effects of the 5-HT_3_ receptor antagonists (e,g., ondansetron and granisetron) in augmentation with SSRIs on OCD-related symptoms [15–17] and recent evidence suggests that ondansetron and tropisetron (other members of the 5-HT_3_ receptor antagonist family) have been successfully used to treat OCD [12, 16–18], no animal OCD models have directly implicated 5-HT_3_ receptors and only two studies have attempted to investigate the efficacy of granisetron for the treatment of OCD[15, 19]. Despite this, the results of these studies supported the potential efficacy and safety of 5-HT_3_ receptor antagonists for the treatment of patients with OCD. Overall, the studies are not conclusive on the actual benefit of 5-HT_3_ antagonists for OCD are not yet totally conclusive [18].

The use of antiemetics in OCD is not unreasonable. In fact, recent research has shown the important role of disgust in the symptoms of OCD. Excessive disgust reactions may cause some of the symptoms of OCD, and in many cases, can even overshadow the anxiety symptoms[20]. One of the models of the pathology of nausea is based on conditioned and learned disgust [21] and some studies showed the superiority of this hypothesis on rats [22]. Moreover, nausea is one of the important adverse effects of serotonin reuptake inhibitors (SSRIs) and it can reduce compliance to medication. Using 5-HT3 blockers, as augmentation therapy maybe the preferred choice especially in cases of drug-induced nausea[23].

Granisetron has a better tolerability profile, a lower drug-drug interaction, no effect on the activity of cytochrome P450, and a longer duration of action amongst other 5-HT_3_ receptor antagonists[15]. Also, it is well-known that sertraline has the lowest drug interaction among SSRIs approved for the treatment of OCD[24]. These benefits make these two drugs suitable choices as augmentation to evaluate their therapeutic effects on patients with OCD.

Given the need to find new options to increase OCD patients’ treatment responsiveness, this study aimed to investigate the additional benefits of granisetron augmentation therapy with sertraline in OCD symptom reduction compared to sertraline alone.

## Methods

### Trial setting and design

A 10-week, double-blind, randomized, placebo-controlled, parallel-group trial was performed at the outpatient clinics of Iran Psychiatric Hospital and Tehran Psychiatric Institute (affiliated with Iran University of Medical Sciences, Tehran, Iran) from April to December 2019.

### Participants

Patients, aged 18–60 years, with a clinical diagnosis of OCD based on DSM-5 criteria, were screened for the study[25]. Those with a diagnosis of moderate-to-severe OCD, defined by a Yale-Brown Obsessive Compulsive Scale (Y-BOCS) score of ≥ 21 were included[26, 27].

The patients attending the clinics were consecutively checked for the entry criteria and recruited until the sample size was achieved. All of the patients enrolled in the study were assessed with a structured clinical interview designed in accordance with the DSM-5 by an expert psychiatrist[25].

The exclusion criteria were: (1) the presence of life-threatening psychiatric symptoms (such as suicidal ideation); (2) comorbid axis I disorders; (3) serious medical or neurological conditions; (such as brain tumors, epilepsy, degenerative disorders, liver failure, cancers, etc.,.) (4) substance dependence (other than caffeine or nicotine); (5) intellectual disability (based on clinical judgment); (6) pregnancy/breastfeeding; (7) contraindication for the use of granisetron or sertraline; (8) history of the previous psychosurgery for OCD; (9) have a complete response with sertraline in their history. During the conduction of the trial, patients were not permitted to participate in any psychotherapeutic treatment. Furthermore, patients were excluded if they used any psychotropic drugs in the last 6 weeks [11, 12].

### Interventions

Eligible participants were randomized to receive either granisetron, 1 mg twice per day, or placebo for 10 weeks. All participants, regardless of group assignment, also received sertraline, 100 mg/day for 4weeks, and then gradually increased to 200 mg/day. To minimize the side effects to the lowest level, the dosage of sertraline increased slowly every week.

### Outcome

Y-BOCS was used for the assessment of patients at baseline and at weeks 0, 4, 8, and 10 of therapy. Y‐BOCS provides a rating scale for the severity of obsessive‐compulsive symptoms [11, 12]. This clinician‐rated scale contains 10 questions, each item rated from 0 (no symptoms) to 4 (extreme symptoms)[27]. The psychometric properties of the Persian version of Y‐BOCS are approved in previous studies [28–30].

The primary outcome of the trial was the difference of total score of the Y-BOCS among the two groups and between the baseline and the end of the trial. The secondary outcome measure was Y-BOCS compulsion and obsession subscale score changes between two groups during the trial period, and also the complete response, partial response, and remission rates, defined as ≥35%, ≥25% decrease in, and ≤16 Y-BOCS total scores [31]. Moreover, adverse effects were monitored every four weeks using a systematic questionnaire and three open questions to include any other side effects not included in the questionnaire [32–34]. In case of observation of any serious adverse effects during the course of therapy, a physician assessed the potential role of the medication in inducing the adverse effects and omitted the patient from the trial.

### Randomization, allocation, concealment, and blinding

Randomization of participants was conducted with a random permuted block method (ratio of 1:1 and blocks of four). The assigned group of each participant was printed consecutively and enveloped in similar in appearance. The allocation was not in reach of the participants or any outcome assessors. The statistical analyzer, randomizer, and outcome assessor each were separate individuals and were blinded to allocation. Also, granisetron and placebo tablets were similar in shape, color, size, and odor.

### Sample size and statistical analysis

With a between-group difference of five points in Y-BOCS score, type I error of 5%, an effect size of 0.25, and power of 80%, using G-power 3.1.9.2 we calculated a sample size of 44 (22 in each group). Considering a drop-out rate of 30% [11, 12], our final sample size was calculated at 58 (29 in each group). IBM SPSS Statistic 23.0.0 (IBM Corporations, Somers, New York, USA) was used for the statistical analysis. Continuous variables were reported as mean ± SD and categorical variables as n (%). Mean differences (MDs) between groups were reported with their 95% continence internals. Fisher’s exact test, or χ2-test was used for the comparison among categorical variables. The independent samples t-test was conducted for the comparison of continuous variable values, respectively. The comparison of Y-BOCS total and subscale score changes in and between groups during the ten-week course of study was achieved by performing a two-factor, repeated-measure analysis of variance (ANOVA). Whenever sphericity of the data could not be assumed using the Mauchly’s test of sphericity, the Greenhouse-Geisser correction for degrees of freedom was used. Score changes from baseline in the participants of each group was examined using the paired sample t-test. A p-value level of ≤0.05 was defined as significant. Missing data was imputed with last observation carried forward (LOCF) method (a form of intention-to-treat method). The missing follow-up visits value was replaced by that participant’s previously observed value.

**Fig. 1:**
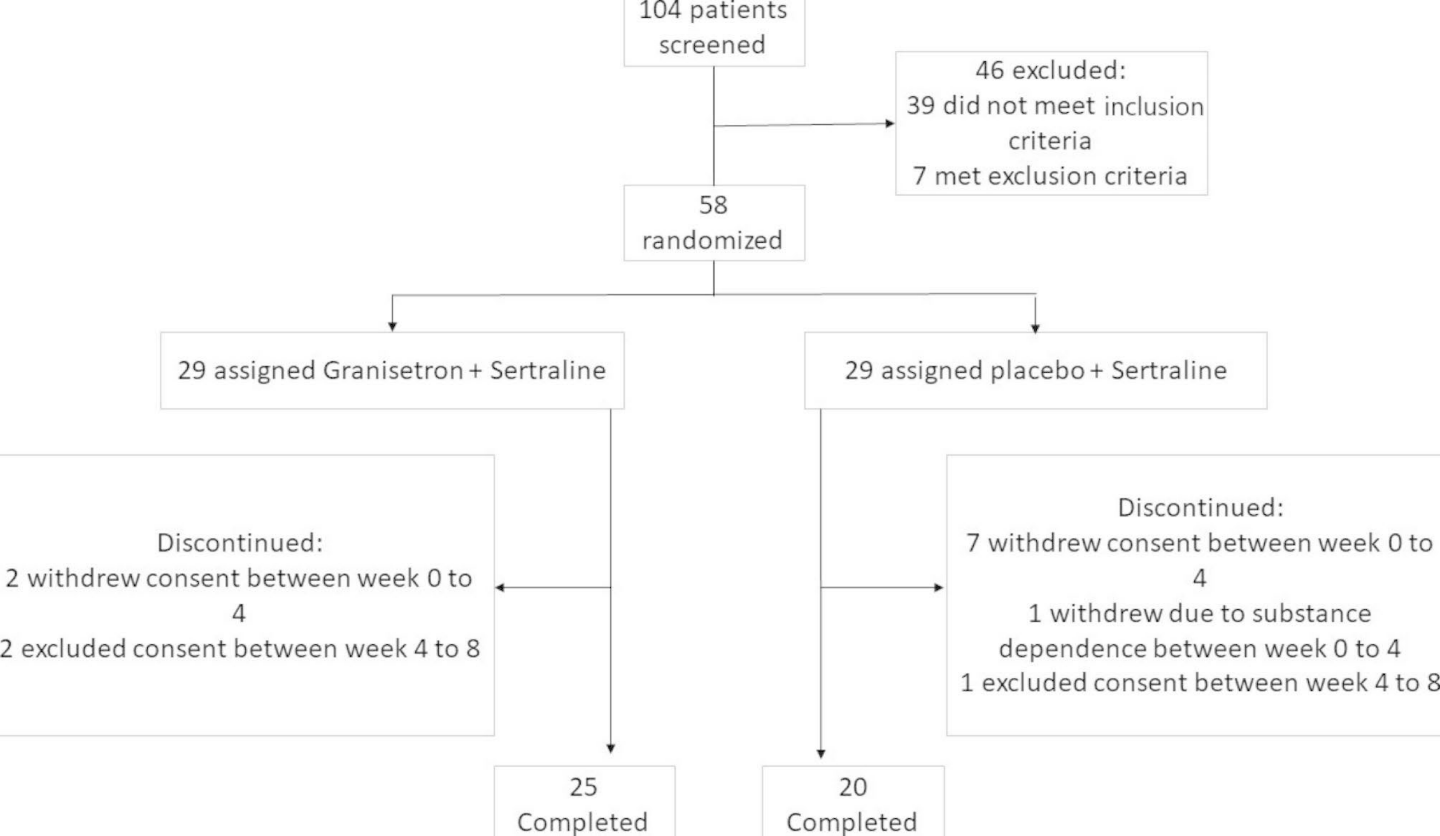
Trial participants’ flow-diagram

## Results

### Participants

One hundred and eight patients were screened primarily, while 58 patients were recruited (randomly assigned to groups of granisetron + sertraline or placebo + sertraline), and 45 patients completed the trial. The trial flow diagram and causes of dropouts are represented in Fig. 1. In the first 4weeks there were 27 patients in the granistron group and 21 patients in the placebo group. The Baseline characteristics are summarized for each group separately in Table 1.

**Table 1:** Baseline characteristics of participants

		Treatment Group				P-value
		granisetron + sertraline (n = 27)		placebo + sertraline (n = 21)		
		Mean ± SD	Count (%)	Mean ± SD	Count (%)	
Age (years)		35.48 ± 10.97		33.44 ± 9.35		0.49
Duration of disease (years)		2.33 ± 3.48		3.44 ± 5.05		0.37
Gender	Female		20 (74.1%)		13 (61.9%)	0.53
	Male		7 (25.9%)		8 (38.1%)	
Education	Illiterate		1 (3.7%)		0 (0.0%)	0.21
	Primary		0 (0.0%)		1 (4.8%)	
	Secondary		2 (7.4%)		1 (4.8%)	
	High school diploma		1 (3.7%)		5 (23.8%)	
	University Education		23 (85.2%		14 (66.7%)	
Marital status	Single		16 (59.3%)		13 (61.9%)	1
	Married		11 (40.7%)		8 (38.1%)	
Employment	Employed		15 (55.6%)		10 (47.6%)	0.46
	Unemployed		7 (25.9%)		3 (14.3%)	
	Housewife		5 (18.5%)		5 (23.8%)	
	Student		0 (0.0%)		3 (14.3%)	
Previous treatment	Yes		12 (44.4%)		12 (57.1%)	0.56
	No		15 (55.6%)		9 (42.9%)	
Y-BOCS score (week 0)	Total	28.92 ± 7.15		27.5 ± 4.95		0.44
	Obsession	16.62 ± 3.04		14.75 ± 3.79		0.06
	Compulsion	12.48 ± 5.41		12.5 ± 3.2		0.98

SD: standard deviation; Y-BOCS: Yale-Brown Obsessive-Compulsive Scale

### Y-BOCS total score

**Fig. 2:**
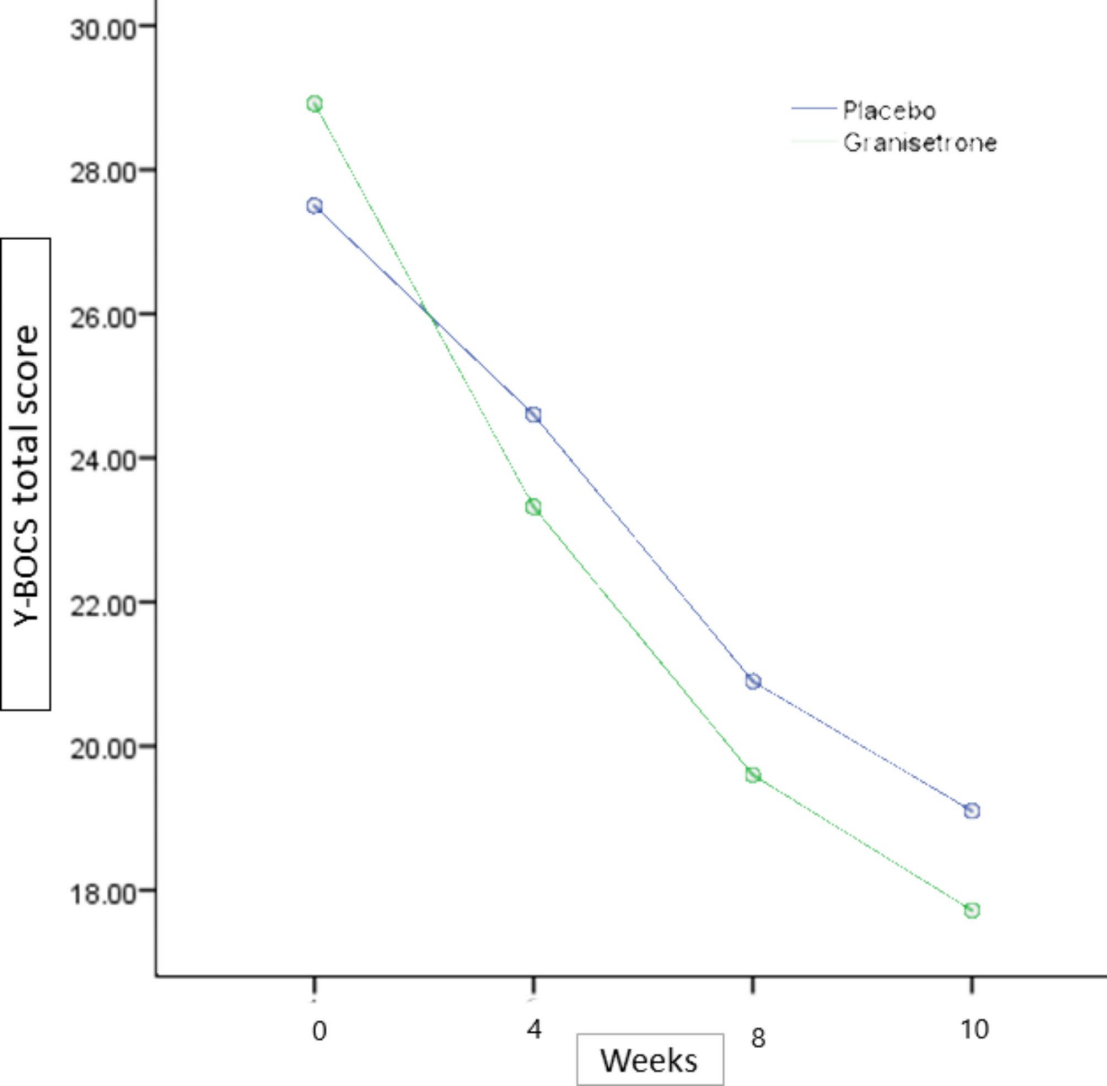
Yale-Brown Obsessive-Compulsive Scale (Y-BOCS) total score trend for each group during the trial course

The baseline Y-BOCS total score was not significantly different between the groups (MD (95% CI) = 1.4(− 2.26,5.10), p-value = 0.442) (Table 1). Total Y-BOCS score changes from baseline in the granisetron group at fourth and tenth week of the study was MD (95% CI) = 5.6 (1.21,9.98) (p-value < 0.01) at week 4 and MD (95% CI) = 11.2 (6.65,15.74) (p-value < 0.01) at the week 10, respectively. In the other hand, participants in the placebo group experienced not significant Y-BOCS total score drop at the week 4(MD (95% CI) = -2.9 (-6.47, 0.67) (p-value = 0.1)), while Y-BOCS total score drop at week 10 /9MD (95% CI) = 8.4 (4.78,12.01) (p-value < 0.01)). In the end, Repeated measures ANOVA revealed no significant difference for the time between granisetron and placebo groups (Greenhouse-Geisser F(2.240,96.307) = 2.31, p-value < 0.09). (Fig. 2) (Table 2).

At the end of the study, no significant difference was observed between the granisetron and the placebo group neither in partial response (20 patients − 80%– vs. 14 − 70%–, p-value = 0.43) nor in remission rate (15 − 60%– vs. 7 − 35%–, p-value = 0.09). Nonetheless, a significant difference was observed in the complete response rate (20 − 80%– vs. 8 − 40%–, p-value = 0.01).

### Y-BOCS obsession subscale score

**Fig. 3:**
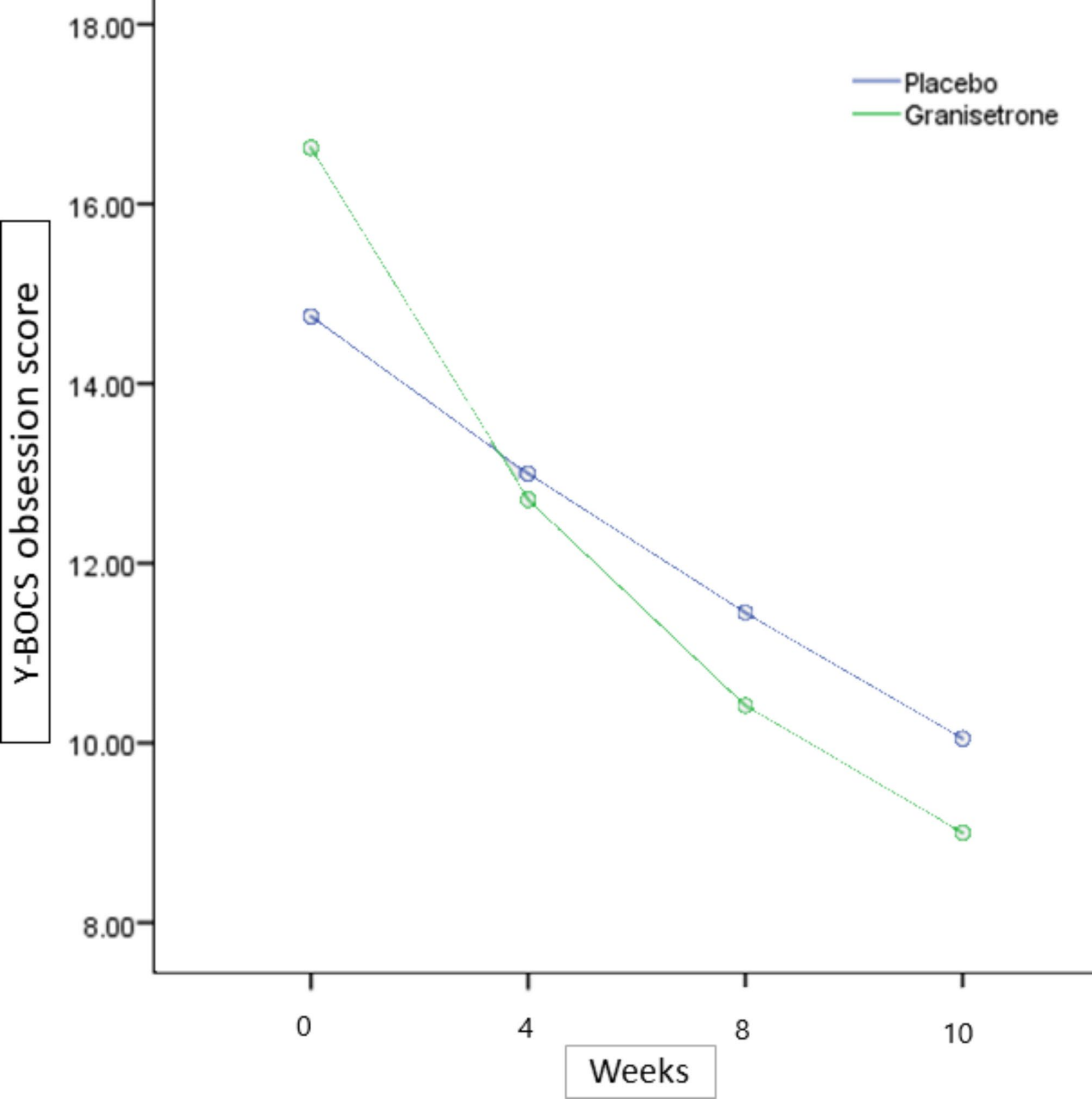
Yale-Brown Obsessive-Compulsive Scale (Y-BOCS) obsession subscale score trend for each group during the trial course

The baseline Y-BOCS obsession subscale score did not significantly differ among treatment groups (MD (95% CI) = 1.87 (− 0.11,3.85), p-value = 0.06)) (Table 1). Obsession Y-BOCS score changes from baseline in the granisetron group at the week4 and the week 10 of the study was MD (95% CI) = 3.92 (1.86,5.97) (p-value < 0.01) at the week 4 and MD (95% CI) = 7.62 (5.40,9.83) (p-value < 0.01) at the week 10, respectively. Participants in the placebo group experienced not significant Y-BOCS total score drop at the week 4 (MD (95% CI) = 1.75 (-0.71,4.21) (p-value 0.15)) but their score change mean differences were MD (95% CI) = 4.7 (2.39,7.00) (p-value < 0.01) at the week 10. The time×treatment group interaction analysis by repeated-measures ANOVA revealed that granisetron group participants significantly experienced higher Y-BOCS obsession subscale score decrease (Greenhouse-Geisser F(2.32,97.57) = 4.52,p-value = 0.01) (Table 2). The Y-BOCS obsession subscale score change trend for each group is presented in Fig. 3.

### Y-BOCS compulsion subscale score

**Fig. 4:**
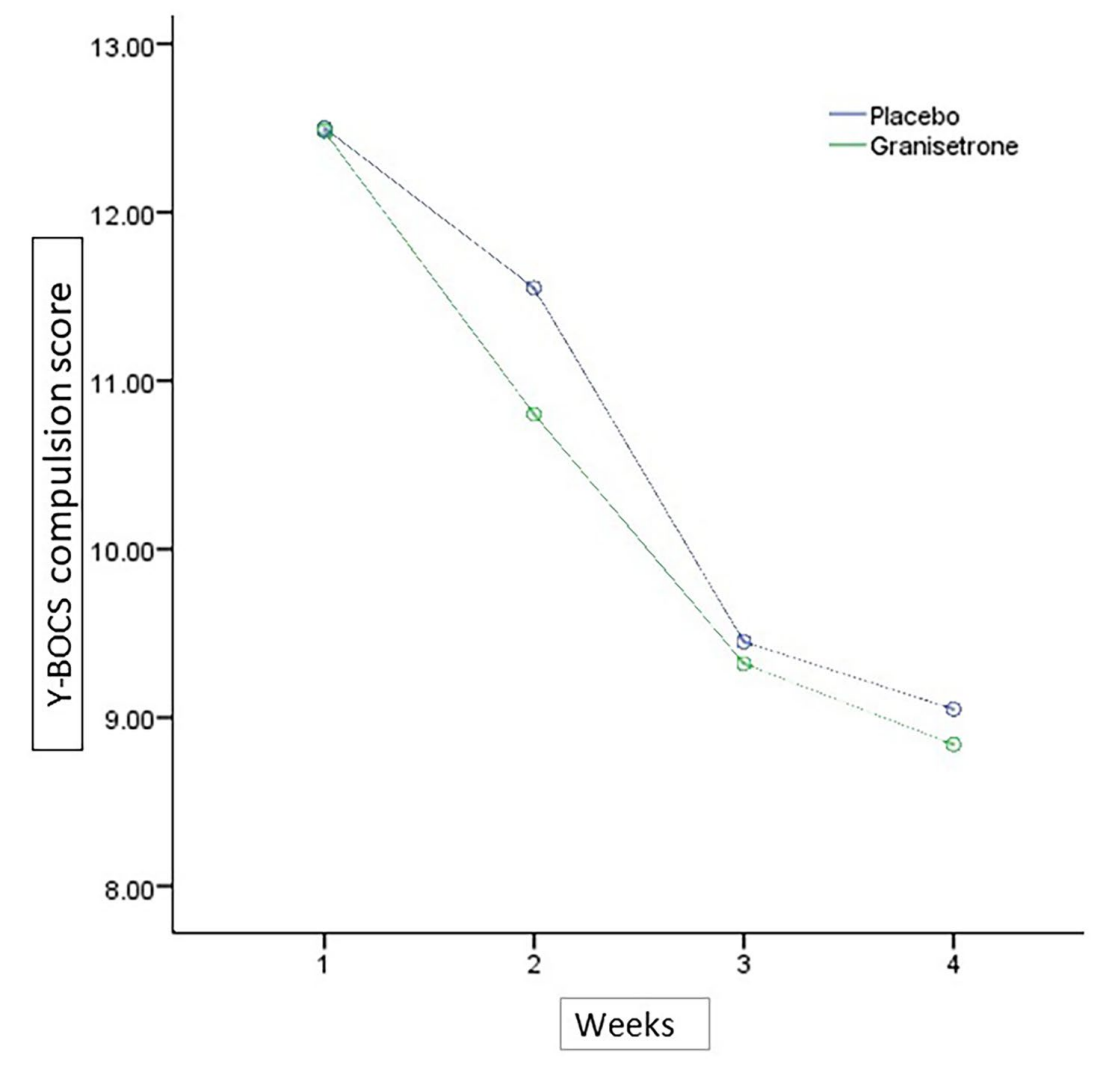
Yale-Brown Obsessive-Compulsive Scale (Y-BOCS) compulsion subscale score trend for each group during the trial course

The baseline Y-BOCS compulsion subscale score did not significantly differ among treatment groups (MD (95% CI) =-0.02 (− 2.7,2.66), p-value = 0.98)) (Table 1). Compulsion Y-BOCS score changes from baseline in the granisetron group at the week 4 was not significant but they experienced a significant drop at the week 10 of the study was MD (95% CI) = 3.64 (0.73,6.54) (p-value 0.01). Similarly, in the placebo group the change from the baseline was not significant in the week 4 but they experienced significant Y-BOCS compulsion score drop at the week 10 (MD (95% CI) = 3.45 (1.22,5.67) (p-value < 0.01)). The time×treatment group interaction analysis by repeated-measures ANOVA revealed no significant difference (Greenhouse-Geisser F(2.13,91.97) = 0.315,p-value = 0.74) (Table 2). The Y-BOCS compulsion subscale score change trend for each group is presented in Fig. 4.

### Adverse effects

Adverse events were recorded during the study. Side effects were mild and did not result in withdrawal. The frequency of side effects was not different between the two groups (Table 3).

**Table 2:** Comparison of Yale-Brown obsessive-compulsive scale (Y-BOCS) subscales score change from baseline for treatment groups

Y-BOCS subscale score reduction		Treatment group					
		granisetron + sertraline			placebo + sertraline		
		Mean ± SD	MD (95% CI)	p-Value	Mean ± SD	MD (95% CI)	p-Value
Obsession	Week 4	12.7 ± 4.36	3.92 (1.86–5.97)	0.0003	13 ± 4.11	1.75 (-0.71-4.21)	0.15
	Week 8	10.41 ± 4.4	6.21 (4.14–8.27)	0.0001	11.45 ± 3.66	3.3 (0.97–5.62)	0.006
	Week 10	9 ± 4.87	7.62 (5.40–9.83)	0.0001	10.05 ± 3.6	4.7 (2.39-7.00)	0.0002
Compulsion	Week 4	10.8 ± 5.15	1.68 (-1.20-4.56)	0.24	11.55 ± 3.8	0.95 (-1.24-3.14)	0.38
	Week 8	4.93 ± 4.86	3.16 (0.35–5.96)	0.02	9.45 ± 3.84	3.05 (0.84–5.25)	0.007
	Week 10	8.84 ± 5.21	3.64 (0.73–6.54)	0.01	9.05 ± 3.91	3.45 (1.22–5.67)	0.003
Total	Week 4	23.32 ± 8.83	5.6(1.21–9.98)	0.013	24.6 ± 6.42	2.9 (-6.47- 0.67)	0.10
	Week 8	19.6 ± 8.55	9.32 (5.01–13.62)	0.0001	20.9 ± 6.19	6.6 (3.09–10.10)	0.0005
	Week 10	17.72 ± 9.34	11.2 (6.65–15.74)	0.0001	19.1 ± 6.51	8.4 (4.78–12.01)	0.001

SD: standard deviation; Y-BOCS: Yale-Brown Obsessive-Compulsive Scale; CI: confidence interval; MD: mean difference;

**Table 3:** Frequency of adverse events in the two groups

Adverse events	Treatment group				
	granisetron + sertraline		placebo + sertraline		
	N	%	N	%	p-value
Muscle pain	1	3.7	0	0	0.99
Headache	1	3.7	0	0	0.99
Diarhea	0	0	1	4.8	0.43
Constipation	1	3.7	0	0	0.99
Decreased libido	3	11.1	1	4.8	0.62
Decreased appetite	1	3.7	0	0	0.99
Lightheadness	1	3.7	1	4.8	0.99
Tremor	1	0	0	0	0.99
Skin lesions	1	0	0	0	0.99
Motor tic	0	0	1	4.8	0.43
Palpitation	1	3.7	0	0	0.99
Insomnia	2	7.4	1	4.8	0.99
Itching	0	0	1	4.8	0.43
Restlesness	0	0	1	4.8	0.43
Vomiting	1	3.7	0	0	0.99

N: number; %: percentage among treatment group

## Discussion

The results showed a greater reduction in Y-BOCS total score, as well as its subscales in patients treated by granisetron plus sertraline in comparison with placebo plus sertraline. However, this difference was statistically significant only for the obsession subscale. Furthermore, our trial showed more partial responses, complete responses, and remission rates among patients receiving granisetron in comparison with placebo. Despite the difference in remission rates between the two groups (60% in the granisetron arm, and 35% in the placebo arm), the only difference in the complete response rate was statistically significant. This result may be explained by the small sample size of our study. Interestingly, our study showed that the rate of observed side effects was not significantly different between the granisetron and placebo groups.

The inclusion of OCD patients scoring at least 21 at the Y-BOCS (moderate to severe OCD), may have caused the statistical insignificance between the two groups. The high score in the baseline caused a large reduction in the absolute scores in both arms and this simultaneous large reduction could alter the significance of change difference between two groups.

The findings of our study are in agreement with previous trials and our hypothesis, which stated granisetron is an effective and well-tolerated agent to be used as adjuvant therapy to SSRIs for the treatment of OCD. To the best of our knowledge, the current study is the first 10-week double-blind randomized placebo-controlled trial to investigate the efficacy and safety of granisetron as adjuvant therapy to sertraline for moderate to severe OCD. In a recent study, Askari et al. reported that granisetron augmentation of fluvoxamine results in a greater reduction in OCD symptoms compared to a placebo[15]. They also reported significantly higher remission and complete response rates among patients in the granisetron group[15]. Our findings are in agreement with their report. Similar to our results, Askari et al. reported the safety and well-tolerability of granisetron plus fluvoxamine in comparison with placebo plus fluvoxamine[15]. However, we believe that our trial has some strengths in comparison with their trial. The key strengths of the present study are its larger sample size and longer duration of follow-up. Moreover, we used sertraline as a standard treatment of OCD for both granisetron and placebo groups. It is well-known that sertraline has the lowest drug-drug interaction among SSRIs approved for the treatment of OCD[24], and it seems to be the best option for adjuvant therapy. In the same vein, Sharafkhani et al. (2019) reported the efficacy of granisetron in patients with treatment-resistant OCD in a 14-week trial in comparison with the ondansetron arm and placebo arm. Although they reported the efficacy of granisetron in the treatment of the patients, they concluded that ondansetron is a superior option to granisetron[19]. The results of their study are hardly comparable to ours because the present study was performed on patients with moderate to severe OCD, not treatment-resistant patients.

Although the evidence presented thus far supports the efficacy and safety of 5HT_3_ receptor antagonists such as ondansetron and tropisetron in the treatment of OCD patients [12, 16, 17], a recent systematic review concluded that more clinical trials are needed to confirm the efficacy of these agents as treatment options for OCD[18].

The exact mechanism of 5HT_3_ receptor antagonists in the treatment of OCD symptoms is not yet well-known. Some researchers have reported that the 5HT_3_ antagonists may ameliorate dopamine hyperactivity in ventral tegmental area in animal models[35], which is one of the known dysregulated systems in OCD, more specifically, due to its importance in reward circuity[36]. Another possible mechanism is the amelioration of dopaminergic activity in prefrontal areas, particularly rich in 5HT3 receptors [37, 38]. The efficacy of dopamine antagonists in the treatment of OCD, which has been shown in previous studies, supports this hypothesis as well[39, 40].

On the other hand, 5HT_3_ antagonists modulate the glutamatergic system through N-methyl d- aspartate (NMDA) receptor antagonism. Recently, augmentation strategies using glutamatergic agents has been explored, with promising results, in patients with OCD [11, 28, 41].

## Limitations

Finally, some important limitations need to be considered. First, a 10week trial for following up OCD patients is a little short and a longer study is suggested. As we thought that the inclusion of moderate to severe OCD patients may have driven the absolute score reduction in both arms and caused an insignificant difference between the two groups, performing other similar studies on patients with lower Y-BOCS scores can be valuable. Also, the rate of score changes that we used to define partial and complete response and remission are higher in some other studies. Using those definitions can affect our results. Additionally, our study was not performed on treatment-resistant OCD patients and the findings cannot be generalized to this population.

## Conclusion

Taken together, these results suggest that granisetron augmentation of sertraline may increase the rate of response in patients with moderate to severe non-refractory OCD. Further studies are suggested in this regard. There is abundant room for further progress in determining the exact mechanism of action of granisetron in the treatment of OCD.

## Data Availability

The datasets generated and/or analysed during the current study are not publicly available due to confidentiality concerns (in the informed consent, we have made a commitment to the participants to publish only the general and group results of the study) but are available from the corresponding author on reasonable request.

## List of abbreviations

Obsessive-Compulsive Disorder: OCD.
N-Methyl-D-aspartate: NMDA.
selective serotonin reuptake inhibitor: SSRI.
Diagnostic and Statistical Manual of Mental Disorders: DSM–5.
Yale-Brown obsessive compulsive scale: Y-BOCS.
exposure and response prevention: ERP.
5-hydroxytryptamine receptor type 3: 5-HT_3_.
analysis of variance: ANOVA.
last observation carried forward: LOCF.
Mean difference: MD.
confidence interval: CI.
standard deviation: SD.
